# *Fuzheng Kang’ai* decoction combined with gefitinib in advanced non-small cell lung cancer patients with epidermal growth factor receptor mutations: study protocol for a randomized controlled trial

**DOI:** 10.1186/s13063-015-0685-2

**Published:** 2015-04-10

**Authors:** Xiao-Bing Yang, Wan-yin Wu, Shun-qin Long, Hong Deng, Zong-Qi Pan, Wen-Feng He, Yu-Shu Zhou, Gui-Ya Liao, Qiu-Ping Li, Shu-Jing Xiao, Jiao-Zhi Cai

**Affiliations:** Department of Oncology, Guangdong Provincial Hospital of Traditional Chinese Medicine, No. 111 Dade Road, Guangzhou, Guangdong 510120 China

**Keywords:** Non-small cell lung cancer, Chinese herbal medicine, *Fuzheng Kang’ai* decoction, Gefitinib, Randomized controlled trial

## Abstract

**Background:**

Patients with advanced non-small cell lung cancer (NSCLC) harboring mutations of the epidermal growth factor receptor (*EGFR*) gene respond well to the *EGFR* tyrosine kinase inhibitor (TKI) gefitinib. Chinese herbal medicine (CHM) has been used as a complementary therapy for cancer for decades in China. CHM was proved to be effective in improving the quality of life (QOL) and reducing the toxicity associated with chemotherapy in patients with NSCLC. The purpose of the present trial is to determine whether CHM (*Fuzheng Kang’ai* decoction (FZKA), a CHM formula) combined with gefitinib results in longer progression-free survival with less toxicity than gefitinib alone.

**Methods/Design:**

This is a randomized, placebo-controlled, double-blind trial. This trial is designed to determine if CHM (FZKA) combined with gefitinib results in longer progression-free survival with less toxicity than gefitinib alone. A total of 70 NSCLC patients with *EGFR* mutations will be randomly assigned to treatment group (gefitinib plus FZKA granules) or control group (gefitinib plus placebo). The primary endpoint is progression-free survival. Secondary endpoints are: (1) overall survival; (2) disease control rate; (3) QOL, measured with the questionnaire of Functional Assessment of Cancer Therapy-lung (FACT-L 4.0) and Lung Cancer Symptom Scale and (4) safety.

**Discussion:**

In previous clinical practice, we found that CHM (FZKA) could improve the therapeutic efficacy of gefitinib. This study will provide objective evidence to evaluate the efficiency of CHM combined with gefitinib in NSCLC patients with *EGFR* mutations, and may provide a novel regimen for patients with NSCLC.

**Trial registration:**

Chinese Clinical Trial Registry (www.chictr.org): ChiCTR-IOR-14005679, registered 17 December 2014.

## Background

Lung cancer is the leading cause of cancer-related mortality worldwide [1]. Non-small cell lung cancer (NSCLC) accounts for 85 to 90% of all cases of lung cancer, and platinum-based doublet chemotherapy is the standard treatment for advanced NSCLC. However, chemotherapy in patients with metastatic NSCLC has reached a therapeutic plateau, with a response rate of less than 35%, and a median overall survival of 6.9 to 11.3 months [2,3].

The discovery of targeted therapy has led to a new era of individualized treatment in lung cancer; epidermal growth factor receptor (*EGFR*) tyrosine kinase inhibitors (TKIs) have been a new paradigm of lung cancer treatment. Gefitinib, a special TKI of *EGFR*, is a representative of first-generation *EGFR*-TKIs which compete with adenosine triphosphate (ATP) in binding to the tyrosine kinase domain. Gefitinib is especially effective in NSCLC patients with *EGFR* mutations, with a response rate of about 70 to 80% [4-6]. *EGFR*-activating mutations are found in the kinase domain of the *EGFR* gene, and comprise mostly of in-frame deletions of exon 19 and L858R substitution in exon 21 [7-9]. Two randomized phase III trials from Japan demonstrated that progression-free survival (PFS) with gefitinib was significantly longer than platinum-based chemotherapy as a first-line treatment in patients with advanced NSCLC who carried activating *EGFR* mutation [10,11]. Although gefitinib results in longer PFS compared with chemotherapy, most patients will eventually develop acquired resistance in 10 to 14 months after treatment [12]. In addition, adverse effects of gefitinib (such as rash, acne or diarrhea) have sometimes resulted in discontinuation of treatment [13].

Chinese herbal medicine (CHM) has been used in China for thousands of years. Some studies have reported that CHM could reduce the occurrence of adverse reactions (such as anaemia and neutropenia) induced by chemotherapy [14]. It is possible that CHM could be used as an adjuvant to chemotherapy for the treatment of NSCLC to improve quality of life (QOL) and compliance to chemotherapy by reducing incidence of anaemia and neutropenia [15]. CHM could also reduce recurrence rate and prolong overall survival [16]. *Fuzheng Kang’ai* decoction (FZKA), a 12-herb Chinese formula, was developed to treat NSCLC based on the theory of combining disease and syndrome with traditional Chinese medicine, and has been used to treat NSCLC in Guangdong Provincial Hospital of Traditional Chinese Medicine for more than 10 years. Our previous retrospective study demonstrated that treatment with gefitinib plus CHM (FZKA) prolonged PFS and median survival time (MST) compared with gefitinib alone in patients with NSCLC [17]. In this study, we aim to assess whether gefitinib plus CHM could prolong PFS or MST in patients with NSCLC compared with gefitinib alone.

## Methods/Design

### Study design

This is a randomized, double-blind, placebo-controlled trial. The study aims to enroll 70 patients with advanced NSCLC who carry activating *EGFR* mutations. Participants are randomized using a ratio of 1:1 to either the treatment group (gefitinib plus FZKA granules) or control group (gefitinib plus placebo). Gefitinib combined with CHM or placebo will be administered until progression of the disease, or development of unacceptable toxicities.

The trial protocol has been approved by the Research Ethical Committee of Guangdong Provincial Hospital of Traditional Chinese Medicine (reference: B2014-059-01). The trial will be conducted in accordance with the Helsinki Declaration [18] and will be monitored by the trial agency at Guangdong Provincial Hospital of Traditional Chinese Medicine.

### Recruitment and consent

**Figure 1:**
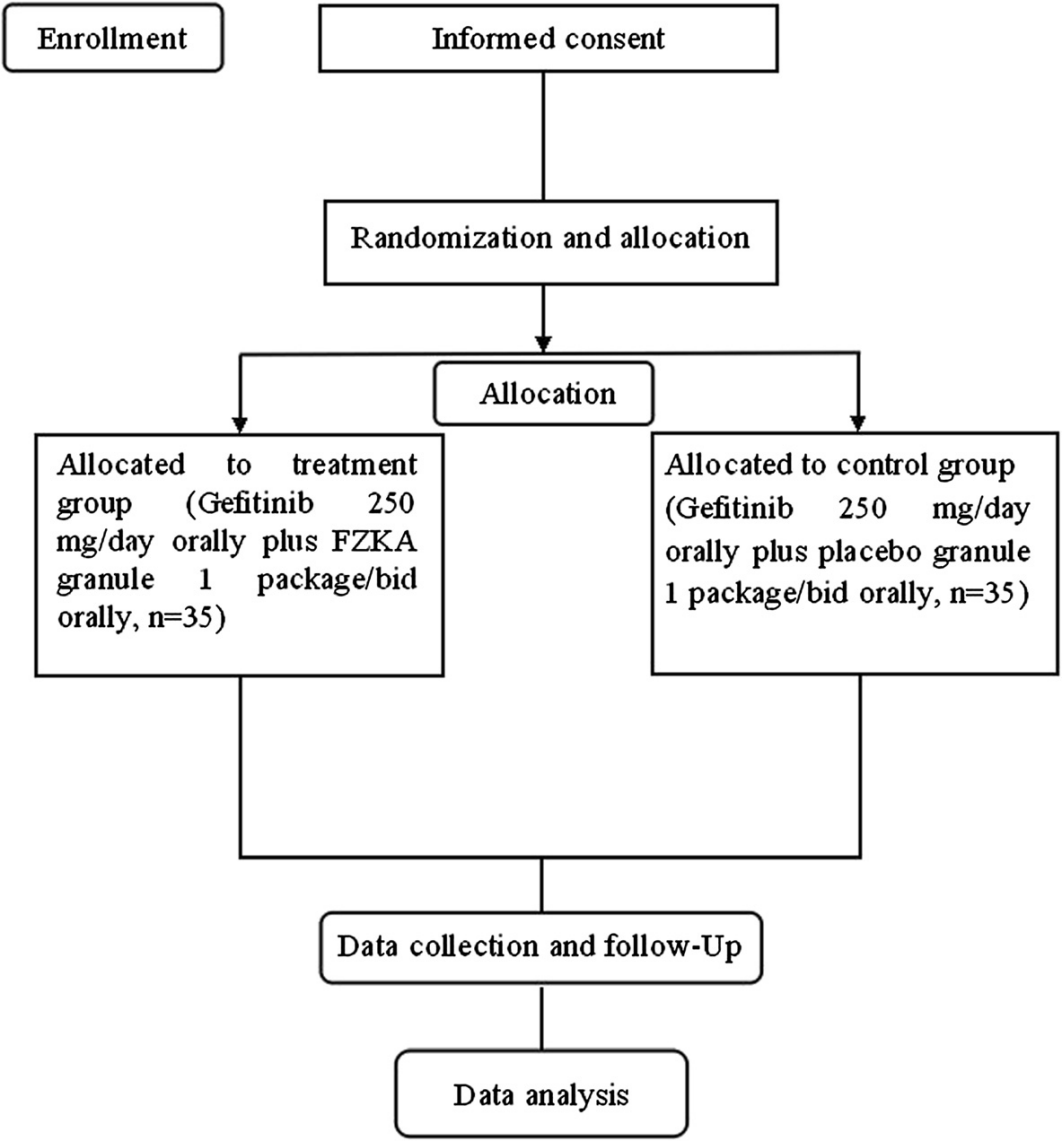
**Study flowchart.** FZKA, *Fuzheng Kang’ai* decoction.

A total of 70 subjects with advanced NSCLC patients carrying exon 19 deletions or L858R mutations will be recruited in Guangdong Provincial Hospital of Traditional Chinese Medicine. All candidates will go through a standardized interview process and receive more information about the study and its treatments. Written consent will be obtained from each patient. The purpose, procedures, potential risks and benefits of the study will also be explained thoroughly to the participants. The participants will be able to withdraw from the study at any time without consequence. The trial will be executed from January 2015 to December 2017, including the enrollment and follow-up periods (Figure 1).

### Inclusion criteria

Participants meeting the following criteria will be included:

1. with histologically or cytologically confirmed stage IIIB or IV NSCLC with histologic features of adenocarcinoma, squamous cell carcinoma or adenosquamous carcinoma;
2. Aged from 18 to 80-years-old;
3. Harboring activating *EGFR* mutations (either exon 19 deletion or L858R in exon 21);
4. Eastern Cooperative Oncology Group (ECOG) performance status score 0 to 2;
5. No previous chemotherapy or biologic or immunologic therapy;
6. Measurable lesion according to the Response Evaluation Criteria in Solid Tumors (RECIST);
7. Adequate organ function;
8. Good compliance and sign a written informed consent.

### Exclusion criteria

Participants meeting one or more of the following criteria will be excluded:

1. Severe hypersensitivity to gefitinib or any component of FZKA;
2. Received previous drug therapy that targeted *EGFR*;
3. Expected life expectancy of less than two months;
4. A history of interstitial lung disease;
5. Any evidence of severe or uncontrolled systemic disease (for example, unstable or uncompensated respiratory, cardiac, hepatic or renal disease);
6. Diagnosed with central nervous system metastasis that has not yet been treated with surgery and/or radiation;
7. Poorly controlled pleural effusion;
8. Pericardial effusion or ascites necessitating drainage;
9. Active double cancer;
10. Currently pregnant or breastfeeding.

### Interventions

Patients in the treatment group will receive gefitinib (250 mg/day orally) plus FZKA granules (1 package/bid orally), while those in the control group will receive gefitinib(250 mg/day orally) plus placebo granules (1 package/bid orally). Gefitinib and FZKA or the placebo will be administered until progression of the disease, development of unacceptable toxicities, or withdrawal of treatment.

The FZKA granules will consist of *Pseudostellaria heterophylla (Miq.) Pax ex Pax et Hoffm. (Taizishen)* 30 g, *Atractylodes macrocephala Koidz. (Baizhu)* 15 g, *Astragalus membranaceus (Fisch.) Bge. (Huangqi)* 30 g, *Oldenlandia diffusa (Willd.) Roxb. (Baihuasheshecao)* 30 g, *Solanum nigrum L. (Longkui)* 30 g, *Salvia chinensis Benth (Shijianchun)* 30 g, *Cremastra appendiculata (D. Don) Makino (Shancigu)* 30 g, *Coix lachrymal-jobi L. (Yiyiren)* 30 g. *Akebia quinata (Thunb.) Decne (Bayuezha)* 30 g, *Rubus parviflolius L. (Shepaole)* 30 g. *Curcuma kwangsiensis S.G. Lee et C.F. Liang (Ezhu)* 15 g, and *Glycyrrhiza uralensis Fisch. (Gancao)* 10 g [19].

All of the above herbal medicines will be provided by Guangdong Kangmei Pharmaceutical Co., Ltd. The FZKA granules used in the trial will be manufactured by the Yifang Pharmaceutical Co., Ltd. (Foshan, Guangdong Province, China) that meets the requirements of Good Manufacturing Practice (GMP). The matching placebo will be produced by the same manufacturer as the FZKA granules and consist of starch with no active ingredients. The colour will be made identical by adding artificial pigment, and the taste will be adjusted by adding an intermediate medicine.

### Outcome measures

The primary outcome is PFS, which will be assessed from the date of randomization to the earliest sign of disease progression, as determined by computed tomography or magnetic resonance imaging according to the RECIST criteria [20], or death from any cause.

The secondary outcomes are MST, objective response rate, disease control rate and safety. MST will be assessed from the date of randomization until 50% of participants are dead from any cause, with the use of methods that are similar to those used for the analysis of PFS. Tumor response will be assessed every six weeks during the first year after randomization, and every two months after the first year, until disease progression. The follow-up time of both groups is two years after recruitment.

### Safety assessments

Participants will be questioned and all adverse events (AEs) will be recorded during the treatment at each visit, and all AEs reported will be analyzed. Routine physical examination, full-blood counts and measurements of blood biochemicals will be measured until study treatment is discontinued. Safety and tolerability will be assessed according to National Cancer Institute Common Terminology Criteria for Adverse Events, version 3.0 [21].

### Randomization and blinding

Random assignment will be performed after consent is obtained by using a computer generated, blocked random-allocation sequence with a 1:1 ratio. Blinding is ensured by using a matched placebo granule, identical in color, size, shape and taste. The quality of the matched trial supplies, such as contents, solubility and bacteria contaminations, should be controlled rigorously according to the GMP standards, and be tested and verified by researchers.

This trial is a double-blind trial. The first level is for the case number corresponding to groups (group A, group B), and the second level is for the group corresponding to intervention (the test and placebo groups). The numbers are kept in opaque sealed envelopes. The double levels of blinding are sealed separately, and given to the leader and the sponsor of the clinical research. Emergency letters are sent to each of the centers, saved with the test drug, and properly preserved until the end of the trial. Treatment assignments are blinded to the patients and investigators (including statisticians) until the entire study is completed.

### Sample size calculation

The sample size of this study is based on the validity of assumptions and past clinical experience. Referring to the results of the IPASS study, the PFS in patients with *EGFR* mutations treated with gefitinib was 9.5 months [22]. In our previous clinical practice, the PFS was 10.5 ± 1.5 months in patients with *EGFR* mutations treated with gefitinib plus FZKA [17]. Using a non-inferiority test, an inspection level alpha = 0.05 and a grasp 1-beta = 0.80, the sample size is 28 cases in each group. The number of patients actually provides less than 90% power, assuming a withdrawal rate of 20% (28/(1–0.2) = 35). Therefore, we will recruit a total of 70 patients, with 35 patients in each group.

### Statistical analysis

The data will be collected and analyzed according to the intention-to-treat principle. Standard statistical techniques will be used to describe characteristics of patients in both groups. We will compare baseline characteristics in both groups, and if the groups are not comparable, we will perform a secondary analysis, adjusting for differences.

PFS and MST will be analyzed by using the Kaplan-Meier method, and compared using the log-rank test. Baseline characteristics will be analyzed using either two-sample t tests or Wilcoxon rank sum tests for continuous data, and chi-squared tests or Fisher’s exact tests for categorical data will be conducted for the purpose of normality. A significance level of 5% will be used throughout the analysis. The incidence rates of AEs will be compared with the use of Fisher’s exact tests. All statistical analysis will be conducted using the SPSS 17.0 statistical package (IBM, Beijing, China). Experts of Guangdong Provincial Hospital of Traditional Chinese Medicine were invited to manage the statistical analysis.

## Discussion

Gefitinib is recognized as a standard first-line treatment for patients with *EGFR* mutations. However, NSCLC patients with *EGFR* mutations who initially respond to gefitinib usually relapse, and the development of secondary resistance inevitably leads to treatment failure. The most common molecular mechanisms of secondary resistance are threonine-to-methionine amino acid change at position 790 (T790M) of the *EGFR* kinase domain (found in 50% of cases), and *MET* amplification (found in up to 20% cases) [23,24]. The toxic effect of gefitinib significantly impacts the QOL in patients with NSCLC, and sometimes causes discontinuation of treatment.

Complementary alternative medicine is becoming more popular all over the world [25]. Traditional Chinese medicine (TCM) is the most popular complementary therapy for cancer patients in China. Among all categories of TCM, CHM is the most commonly used, and has been widely adopted by cancer centers of Asian countries [26,27]. One study reported that CHM could decrease the incidence of chemotherapy-induced muscle pain and reduce symptom severity [28]. CHM may have synergistic effects when combined with chemotherapy or radiotherapy. It is still unknown whether CHM could improve the effect of TKI and reduce its toxicities.

In our previous retrospective study, we found that treatment with gefitinib plus CHM (FZKA) prolonged PFS and MST compared with gefitinib alone in patients with NSCLC. PFS in patients treated with gefitinib plus CHM was 13.1 months compared with 11.43 months in patients treated with gefitinib alone. MST in patients treated with gefitinib plus CHM was 22.83 months compared with 18.7 months in patients treated with gefitinib alone. The incidence of rash in NSCLC patients treated with gefitinib plus CHM (FZKA) was lower than those treated with gefitinib alone. These findings indicate that FZKA may have a toxicity reducing and efficacy enhancing effect on gefitinib [17].

As our previous study was a retrospective study, a well-designed randomized controlled trial is needed to verify the efficacy of CHM (FZKA) plus gefitinib in patients with NSCLC. If this trial provides high-quality evidence for the efficacy and safety of CHM plus gefitinib, it will provide useful clinical information for NSCLC, especially for reducing the toxicity and improving the efficacy of gefitinib. Moreover, it may also provide evidence for optimizing and promoting integrated CHM combined with Western medical treatment in NSCLC.

## Trial status

Recruitment will commence in January 2015, and the trial is scheduled to end in December 2017.

## Abbreviations

AEs: Adverse events
CHM: Chinese herbal medicine
*EGFR*: Epidermal growth factor receptor gene
FZKA: *Fuzheng Kang’ai* decoction
GMP: Good manufacturing practice
MST: Median survival time
NSCLC: Non-small cell lung cancer
PFS: Progression-free survival
RECIST: Response evaluation criteria in solid tumors
TCM: Traditional Chinese medicine
TKIs: Tyrosine kinase inhibitors
